# Supplementary material for: Interleukin-35 Prevents the Elevation of the M1/M2 Ratio of Macrophages in Experimental Type 1 Diabetes
Source: Int J Mol Sci. 2022 Jul 19;23(14):7970. doi: 10.3390/ijms23147970 (PMC9320761; doi:10.3390/ijms23147970)
Supplement: Supplementary file 1 [file ijms-23-07970-s001.zip › ijms-1826792-supplementary.pdf]

Supplementary figures 1-3

Interleukin-35 prevents elevation of the M1/M2 ratio of macrophages in experimental type 1 diabetes

Zhengkang Luo[1], Charlotte Soläng[1], Rasmus Larsson[1] and Kailash Singh [1,*]

*Correspondence: Kailash.Singh@mcb.uu.se; Tel.: +46-184714411

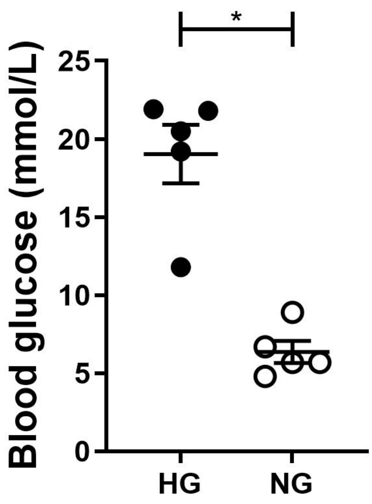

Supplementary Figure S1. Blood glucose levels.

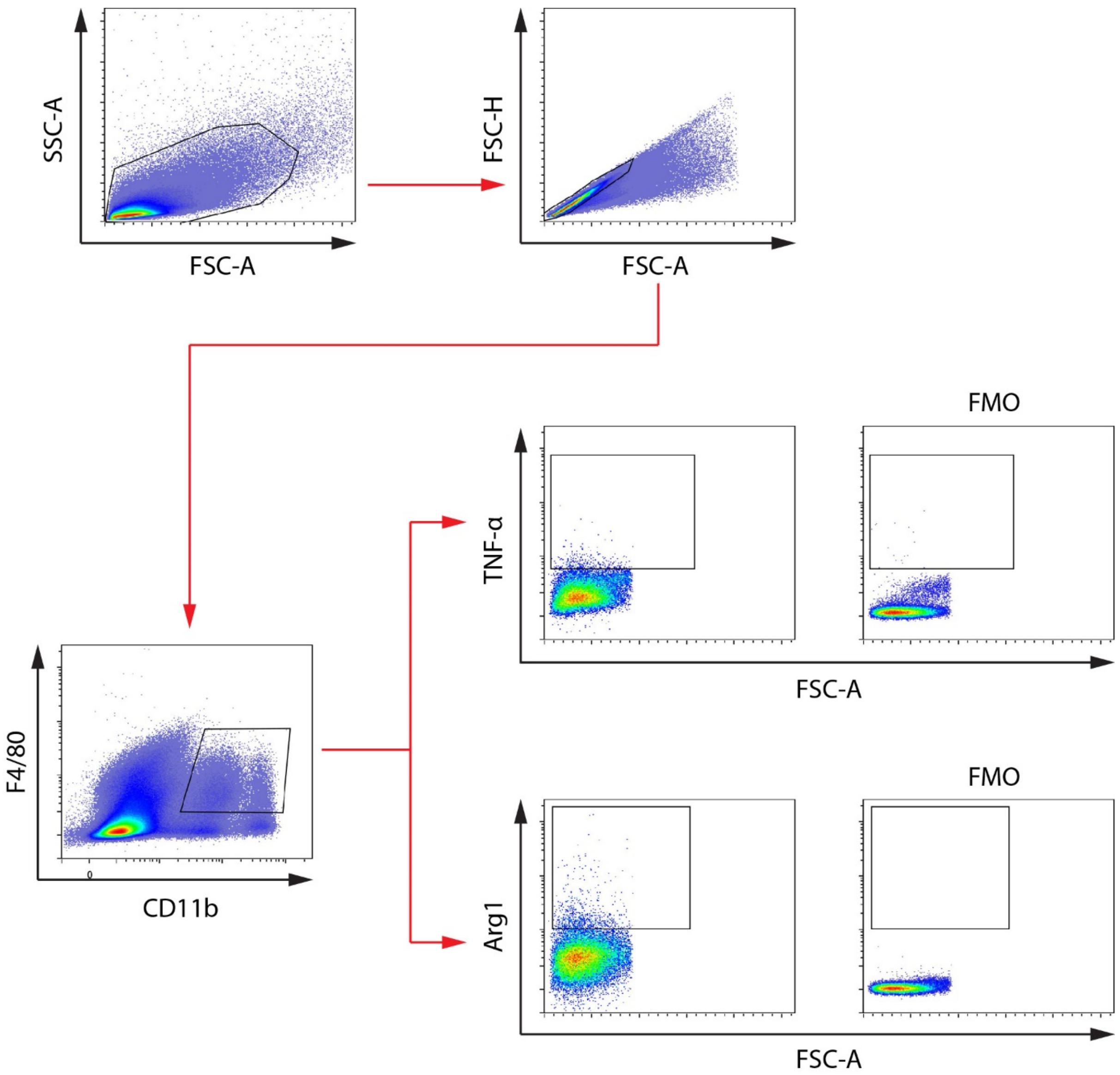

Supplementary Figure S2. Representative gating strategies for macrophages. The live cells were gated based on forward scatter (FSC) and side scatter (SSC). Cells were further gated for CD11b and F4/80 expression. $CD11b^{+}F4/80^{+}$ cells were thereafter gated for the expression of TNF-α and Arg1. Gates were drawn by using fluorescence minus one controls to analyze TNF-α^{+} and $Arg1^{+}$ cells.

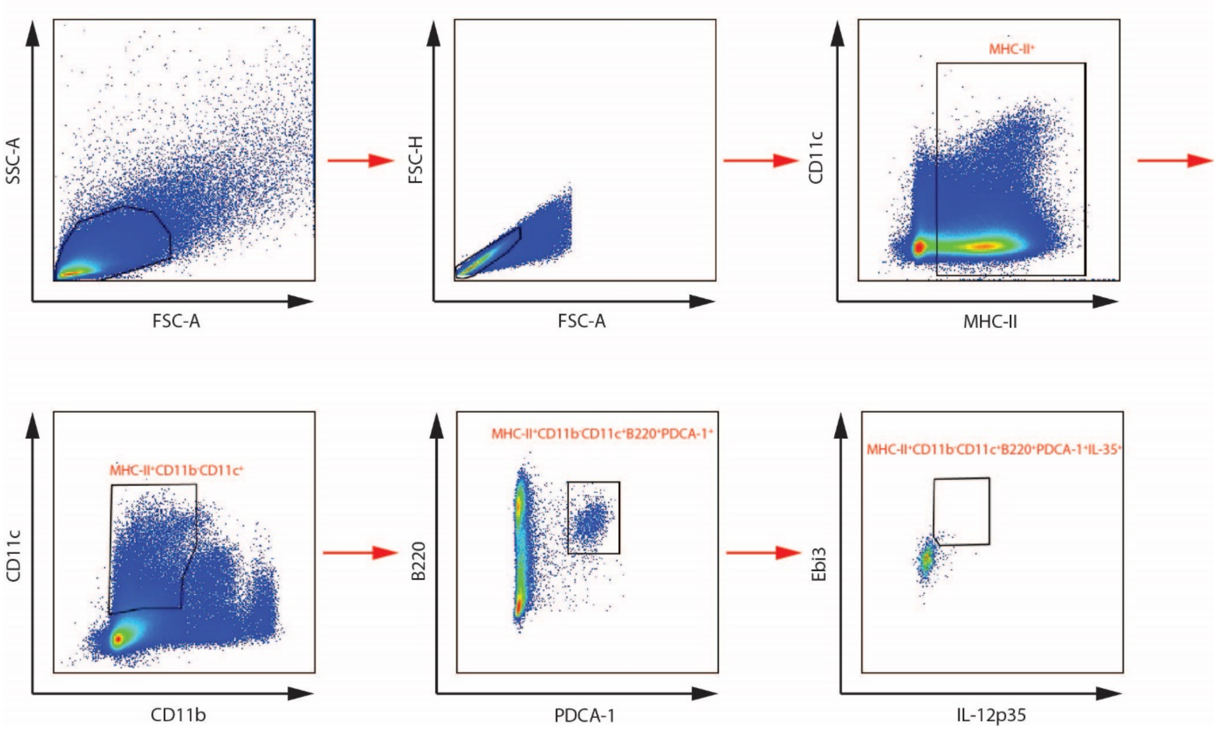

Supplementary Figure S3. Representative gating strategies for pDCs. The live cells were gated based on forward scatter (FSC) and side scatter (SSC). MHC-II$^+$CD11b$^-$ CD11c$^+$B220$^+$PDCA-1$^+$ cells were gated for IL-35 expression (Ebi3 and IL-12p35)
